# Higher plasma omega-6 fatty acids are associated with lower dementia risk: evidence from NMR metabolomics and Alzheimer's disease polygenic risk in the UK Biobank

**DOI:** 10.3389/fpubh.2026.1816467

**Published:** 2026-05-14

**Authors:** Li Luo, Siyin Gong, Dan Liu, Kunyan Zhou

**Affiliations:** 1 Department of Obstetrics and Gynecology, West China Second University Hospital, Sichuan University, Chengdu, China; 2 Key Laboratory of Birth Defects and Related Diseases of Women and Children (Sichuan University), Ministry of Education, Chengdu, China; 3 Department of Neurology, The Second Affiliated Hospital of Chongqing Medical University, Chongqing, China; 4 Department of Ultrasonic Medicine, West China Second University Hospital of Sichuan University, Chengdu, China

**Keywords:** Alzheimer's disease, dementia, NMR metabolomics, omega-3, omega-6, polygenic risk score, polyunsaturated fatty acids, UK Biobank

## Abstract

**Background:**

Circulating polyunsaturated fatty acids (PUFAs) have been implicated in neurodegenerative processes, yet epidemiological evidence remains inconsistent. Genetic susceptibility to Alzheimer's disease (AD), commonly indexed by a polygenic risk score (PRS), is a major determinant of dementia risk. However, few large-scale prospective studies have jointly examined plasma fatty acid profiles and genetic liability to AD in relation to incident dementia.

**Objectives:**

To investigate the independent and combined associations of plasma omega-3 PUFAs, omega-6 PUFAs, and the omega-6/omega-3 ratio with incident dementia, and to determine whether these associations vary according to AD-PRS.

**Methods:**

We analyzed data from 81,827 UK Biobank participants with baseline nuclear magnetic resonance (NMR) metabolomics and genetic information. Plasma fatty acids were quantified using standardized NMR spectroscopy, and genetic risk for AD was assessed using the Enhanced AD-PRS. Incident dementia was identified through linked hospital admission and death registry records. Multivariable-adjusted hazard ratios (HRs) were estimated using Cox proportional hazards models. Dose–response relationships and both additive and multiplicative interactions with AD-PRS were evaluated, alongside subgroup and sensitivity analyses.

**Results:**

Over a median follow-up of 12.1 years, 1,335 participants developed dementia. Higher plasma omega-6 concentrations were consistently associated with a lower risk of dementia, corresponding to an approximately 15% risk reduction per 1 mmol/L increase (HR 0.85, 95% CI 0.79–0.93, *P* < 0.001). In contrast, plasma omega-3 levels (HR 0.97, 95% CI 0.77–1.23, *P* = 0.81) and the omega-6/omega-3 ratio (HR 0.96, 95% CI 0.85–1.09, *P* = 0.55) were not significantly associated with dementia risk. AD-PRS was a strong predictor of incident dementia; however, no statistically significant interactions were observed between AD-PRS and any fatty acid biomarker. Joint analyses suggested only modest attenuation of absolute genetic risk at higher omega-6 concentrations. Findings were robust across multiple sensitivity analyses.

**Conclusions:**

Higher plasma omega-6 PUFA levels were independently associated with a clinically meaningful reduction in dementia risk, whereas omega-3 PUFAs and the omega-6/omega-3 ratio were not. Although elevated omega-6 modestly reduced absolute risk among individuals with high genetic susceptibility to AD, no meaningful gene–nutrient interaction was detected. Further mechanistic and interventional studies are warranted to elucidate underlying pathways.

## 1 Introduction

With rapid global population aging, dementia, including Alzheimer's disease (AD) and vascular dementia, has emerged as one of the fastest-growing causes of morbidity and mortality worldwide, imposing profound burdens on functional independence, family caregiving, and healthcare systems (1, 2). In the absence of disease-modifying therapies, identifying modifiable metabolic and lifestyle determinants that can delay onset or reduce dementia risk has become an urgent public health priority.

Polyunsaturated fatty acids (PUFAs), comprising the omega-3 (n-3) and omega-6 (n-6) families, are integral to brain structure and function. They maintain neuronal membrane integrity, regulate synaptic plasticity, modulate neuroinflammatory responses, and support cerebral energy metabolism (3, 4). Docosahexaenoic acid (DHA), a principal n-3 PUFA, is a key structural component of synaptic membranes, whereas linoleic acid (LA) and arachidonic acid (AA), the predominant n-6 PUFAs, contribute to inflammation–resolution pathways and neuronal repair processes (5, 6). These biological roles implicate PUFAs in multiple mechanisms underlying dementia pathogenesis.

Observational evidence has consistently linked higher omega-3 intake or higher circulating omega-3 levels, particularly DHA, to lower risks of cognitive decline and dementia (7, 8). A meta-analysis involving 103,651 participants reported that greater omega-3 consumption was associated with an approximately 20% reduction in dementia risk (7). In contrast, randomized controlled trials have largely failed to demonstrate cognitive benefits of long-term omega-3 supplementation or disease-modifying effects in AD (9, 10). These discordant findings may reflect limited statistical power, insufficient intervention duration, heterogeneity in baseline nutritional status, or initiation of supplementation beyond a critical preclinical window.

Evidence for omega-6 fatty acids is more limited and relatively inconsistent. Omega-6 PUFAs comprise a heterogeneous group of metabolites, ranging from LA and AA to downstream lipid mediators such as prostaglandins and leukotrienes, which exert both pro-inflammatory and inflammation-resolving effects (11). Epidemiological studies relying on total omega-6 measures may obscure subtype-specific associations (12). Some prospective cohort studies have reported that higher circulating omega-6 levels are associated with slower cognitive decline or reduced dementia risk (13, 14). Conversely, mechanistic studies have suggested that excessive omega-6 metabolism or a high n-6/n-3 ratio may promote neuroinflammation and neurodegeneration (15). Ecological analyses further indicate higher dementia incidence in populations with greater omega-6 intake (16). Furthermore, a recent Mendelian randomization study found no significant association between genetically predicted n-6 PUFA levels and AD risk (LA: OR per 1 SD ≈ 0.98; AA: OR ≈ 1.01) (17). These inconsistencies likely reflect methodological limitations, including dietary recall bias, metabolic heterogeneity across PUFA subtypes, population-specific dietary patterns, and limited follow-up in observational cohorts (12, 18).

Several important gaps therefore remain. Most epidemiological studies have relied on dietary questionnaires, which are prone to measurement error and may poorly capture long-term metabolic exposure (8, 19). Biomarker-based assessments, such as plasma fatty acid profiling or NMR-based lipidomics, provide more objective and physiologically relevant measures for PUFA exposure yet prospective studies using these approaches remain scarce. Existing biomarker studies often focus on either omega-3 or omega-6 fatty acids in isolation, precluding direct comparison or joint evaluation (7, 8). Even fewer studies have incorporated genetic susceptibility, a major determinant of dementia risk, into these analyses (20).

Although genetic liability substantially influences dementia risk, evidence on whether PUFA exposures modify this risk is minimal. There are, however, biological reasons to suspect an interaction. AD-related genetic variants, particularly in the APOE region, influence systemic and cerebral lipid metabolism, including the transport and incorporation of PUFAs into neuronal membranes (15). Concurrently, omega-3 and omega-6 fatty acids regulate neuroinflammation, synaptic plasticity, and membrane integrity—pathways also affected by genetic risk. Therefore, it is plausible that circulating PUFA levels could modify the impact of genetic susceptibility on dementia. Although healthy lifestyle factors may may partially mitigate the effects of high genetic risk (21), no large-scale prospective cohort has systematically examined interactions between PUFA biomarkers and Alzheimer's disease polygenic risk scores (AD-PRS). Hence, the extent to which fatty acid metabolism and genetic predisposition jointly shape dementia risk remains unclear.

To address these gaps, we used standardized NMR-based lipidomic data from the large, prospective UK Biobank cohort to examine the associations of plasma omega-3 and omega-6 fatty acids, and their ratio, with incident dementia. By incorporating an AD-PRS, we further assessed the independent and joint contributions of PUFA biomarkers and genetic susceptibility to dementia risk. We hypothesized that higher circulating omega-6 and omega-3 fatty acids might be associated with lower dementia risk, and that these associations could vary according to genetic risk.

By clarifying the interplay between fatty acid metabolism and genetic liability, this study aims to provide more robust epidemiological evidence to inform targeted strategies for dementia prevention.

## 2 Methods

### 2.1 Study population and data collection

This study retrieved data from the UK Biobank, a large, population-based prospective cohort that recruited over 500,000 participants aged 40–69 years between 2006 and 2010. Participants were enrolled at 22 assessment centers across England, Wales, and Scotland, where they completed touchscreen questionnaires, provided biological samples, and underwent standardized physical examinations (22). Baseline assessments collected extensive information on sociodemographic characteristics, lifestyle behaviors, medical history, and physiological measurements. Detailed study procedures are publicly available (www.ukbiobank.ac.uk). The UK Biobank received ethical approval from the National Health Service and the National Research Ethics Service (REC reference: 21/NW/0157). All participants provided electronic informed consent, and data from participants who subsequently withdrew were removed from the dataset. This study was conducted in accordance with the Strengthening the Reporting of Observational Studies in Epidemiology (STROBE) guidelines.

**Figure 1:**
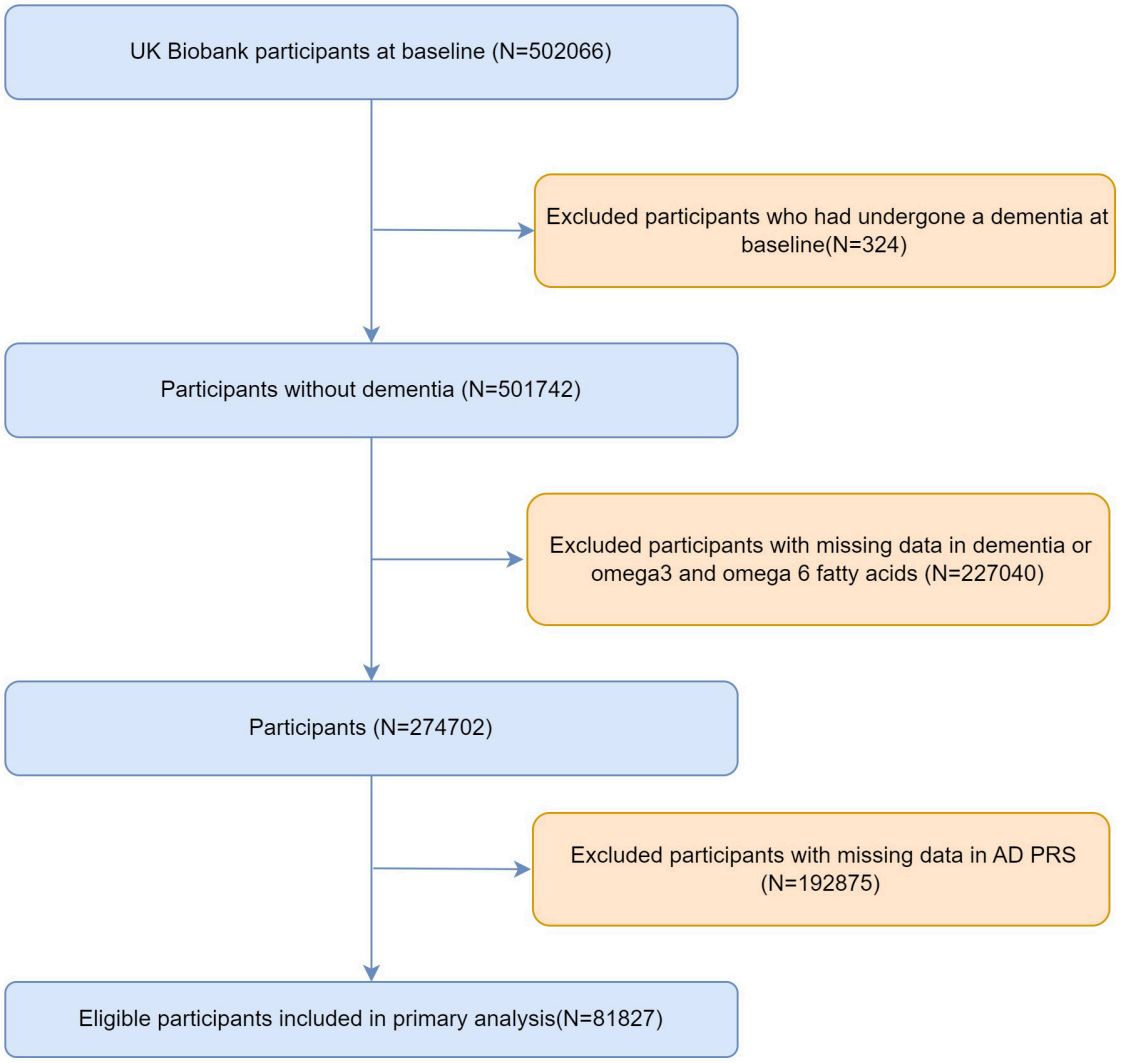
Study participant flow. All-cause dementia (including non-AD dementias) was the primary outcome.

A total of 502,066 individuals were initially recruited. We excluded 324 participants with a diagnosis of dementia at baseline, 227,040 participants with missing plasma omega-3 or omega-6 measurements from NMR metabolomics, and 192,875 participants without available AD-PRS data. The final analytic sample comprised 81,827 participants (Figure 1).

For continuous covariates with missing values, mean imputation was applied. Specifically, the Townsend Deprivation Index (TDI) had 108 missing values (0.13%) and body mass index (BMI) had 415 missing values (0.51%). For categorical variables, missing values were coded as a separate “missing” category, including physical activity level (*n* = 19,607, 23.96%), education level (*n* = 16,045, 19.61%), household income (*n* = 13,512, 16.51%), ethnicity (*n* = 1,205, 1.47%), smoking status (*n* = 663, 0.81%), and alcohol consumption (*n* = 464, 0.57%). This approach allowed all 81,827 participants to be retained in the final analyses.

### 2.2 Exposure assessment

Plasma fatty acid biomarkers were quantified using high-throughput NMR spectroscopy performed by Nightingale Health Ltd. The metabolomics platform measured 249 EDTA-plasma biomarkers across two assessment phases involving randomly selected subsets of the UK Biobank cohort. Of note, plasma samples in the UK Biobank were not collected under fasting conditions, which may introduce some variability in fatty acid measurements. The primary exposures of interest were plasma omega-3 fatty acids (Field ID 23444), omega-6 fatty acids (Field ID 23445), and the omega-6/omega-3 ratio (Field ID 23459), all expressed in mmol/L. These biomarkers provide objective indicators of long-term dietary fatty acid intake and endogenous lipid metabolism. For categorical analyses, omega-3, omega-6, and the omega-6/omega-3 ratio were categorized into tertiles (Q1–Q3). Detailed cutpoints and descriptive statistics are provided in the Supplementary Methods. A complete list of NMR field IDs used in this study is shown in Supplementary Table S1.

### 2.3 Polygenic risk score calculation

Genetic susceptibility to dementia was assessed using the UK Biobank “enhanced” Alzheimer's disease polygenic risk score (AD-PRS; Field ID 26207). This score was centrally generated using a Bayesian PRS framework trained on large-scale meta-analyzed external GWAS and further optimized using UK Biobank-specific GWAS data. The AD-PRS, as provided by UK Biobank, is ancestry-adjusted and standardized to a mean of approximately 0 and a standard deviation of 1. It is genome-wide, includes the APOE region, and represents a weighted sum of AD-associated risk alleles. (https://biobank.ndph.ox.ac.uk/crystal/field.cgi?id=26207). To further minimize residual population stratification, all Cox proportional hazards models were adjusted for the first 20 genetic principal components (PCs), in accordance with UK Biobank quality-control recommendations.

Although the UK Biobank does not provide a PRS for all-cause dementia, AD accounts for approximately 60%−70% of dementia cases. Moreover, prior studies have shown that the AD-PRS strongly predicts incident dementia independently of APOE and other established risk factors (23). Therefore, the AD-PRS was considered an appropriate proxy for overall genetic liability to dementia. As a sensitivity analysis, we additionally examined clinically diagnosed AD as a separate outcome.

AD-PRS was categorized into quintiles and further grouped into three categories (low:Q1, intermediate: Q2–Q4, high: Q5) to facilitate interpretation and risk stratification, consistent with previous UK Biobank studies (23, 24).

### 2.4 Outcome measures

Incident all-cause dementia was identified through linkage to national hospital inpatient records and death registries within the UK Biobank. Dementia diagnoses were ascertained using ICD-9 and ICD-10 codes for AD and other major dementia subtypes. Participants with prevalent dementia at baseline were excluded. A previous validation study demonstrated that these sources have positive predictive values of 80~87% for all-cause dementia, indicating good diagnostic accuracy (25). Follow-up time was calculated from the baseline date to the earliest occurrence of dementia diagnosis, death, or the end of follow-up (December 31, 2021). The ICD codes used to define all-cause dementia outcomes are provided in Supplementary Table S2.

### 2.5 Covariates

Covariates were selected *a priori* based on prior literature as potential confounders and included sociodemographic characteristics, lifestyle factors, and pre-existing comorbidities (7, 14, 18). Age was calculated using the participant's date of birth and baseline assessment date. Socioeconomic status was assessed using the TDI and self-reported household income. Gender (“male,” “female”), ethnicity (“white,” “other”), education attainment (“degree or professional qualification,” “no degree or professional qualification”), smoking status (“never,” “previous,” “current”), and alcohol consumption (“never,” “previous,” “current”) were obtained from the touchscreen questionnaire. BMI (kg/m^2^) was calculated using directly measured height and weight. Physical activity was assessed using a questionnaire adapted from the short form of the International Physical Activity Questionnaire (IPAQ) (26). Total Metabolic Equivalent of Task (MET)-minutes per week were computed by weighting vigorous, moderate, and walking activities according to their respective energy expenditures. Participants were classified as having low ( ≤ 1,200 MET-minutes/week) or high (>1200 MET-minutes/week) physical activity levels (23, 26).

Comorbidities, including hypertension, diabetes mellitus (DM), and stroke, were identified from hospital inpatient records using ICD-9 and ICD-10 codes. Participants with diagnoses recorded prior to baseline were classified as having pre-existing conditions. UK Biobank field IDs for all covariates are provided in Supplementary Table S1, and detailed ICD codes are listed in Supplementary Table S2.

### 2.6 Statistical analysis

Baseline characteristics were summarized according to plasma omega-6 fatty acid levels. Continuous variables were presented as mean ± standard deviation (SD), while categorical variables were expressed as counts (percentages). Cox proportional hazards regression models were used to estimate hazard ratios (HRs) and 95% confidence intervals (CIs) for the associations between plasma fatty acid concentrations and incident dementia. Three models were fitted sequentially: crude model: no covariate adjustment. Model 1: adjusted for age, sex, ethnicity, education level, and TDI. Model 2: additionally adjusted for household income, BMI, smoking status, alcohol consumption, physical activity level, and comorbidities (hypertension, DM, and stroke). The proportional hazards assumption was assessed using Schoenfeld residuals, and no violations were observed for any exposure or for the global models (all *p*-values >0.05; Supplementary Table S3).

To examine potential non-linear associations between omega fatty acid levels and dementia risk, restricted cubic spline (RCS) models with four knots (5th, 35th, 65th, 95th percentiles) were fitted based on the fully adjusted model (Model 2). RCS curves were used to flexibly model and visualize dose–response relationships.

Joint analyses were conducted to evaluate the combined associations of plasma omega fatty acid levels and genetic susceptibility. Participants were cross-classified by tertiles of omega fatty acid concentration and AD-PRS categories (low, intermediate, high). Individuals with both low omega fatty acid levels and low genetic risk served as the reference group. HRs and 95% CIs for joint exposure category were estimated using fully adjusted Cox models.

Additive interactions were quantified using the relative excess risk due to interaction (RERI) and the attributable proportion (AP), calculated following established methods (Li & Chambless, 2007). Confidence intervals for RERI and AP were derived using 1,000 bootstrap replications. Additive interaction was considered absent when the 95% CI of RERI or AP included zero. Multiplicative interactions were evaluated by including cross-product terms between fatty acid levels and stratifying variables (sex, smoking status, alcohol consumption, hypertension, DM, stroke, and AD-PRS) in the Cox models.

Multiple sensitivity analyses were conducted to evaluate the robustness of findings. First, primary analyses were repeated in the larger cohort with available omega fatty acid measurements to evaluate potential selection bias related to missing AD-PRS data. Second, participants who developed dementia within the first three years of follow-up were excluded to reduce potential reverse causation. Third, Fine-Gray subdistribution hazard models were used to account for competing risks from non-dementia mortality. Fourth, Cox models were re-estimated using age as the underlying time scale, with left truncation at baseline age. Fifth, models were additionally adjusted for the alternative fatty acid biomarker (e.g., omega-6 adjusted for omega-3) or mutually adjusted for AD-PRS when interaction terms were not included. Sixth, analyses were repeated after excluding participants younger than 60 years at baseline. Seventh, complete-case analyses were conducted excluding individuals with missing covariate data. Finally, AD was examined as a separate endpoint to assess the specificity of associations relative to all-cause dementia. Given that the primary analyses were hypothesis-driven and focused on a limited number of predefined exposures, no formal adjustment for multiple comparisons was applied, consistent with common practice in hypothesis-driven epidemiological studies.

All statistical analyses were conducted on the UK Biobank Research Analysis Platform using R (version 4.4.1). Statistical significance was defined as a two-sided *P*-value < 0.05.

## 3 Results

### 3.1 Baseline characteristics of the study population

After applying the exclusion criteria (Figure 1), a total of 81,827 participants were included in the analytic cohort, of whom 54.8% were women (*n* = 44,871) and 45.2% were men (*n* = 36,956). Over a median follow-up of 12.1 (IQR: 11.3–12.8) years, 1,335 participants (1.63%) developed incident dementia. Table 1 summarizes baseline characteristics according to tertiles of plasma omega-6 fatty acid concentrations. Participants in the highest tertile were slightly older (56.51 ± 7.75 vs. 55.34 ± 8.77) and were more likely to be women (63.81% vs. 44.51%) compared to those in the lowest tertile. Higher omega-6 fatty acid levels were also associated with healthier lifestyle profiles, including a lower prevalence of current smoking, higher levels of physical activity, and more favorable socioeconomic indicators, such as higher household income and greater educational attainment. The prevalence of major comorbidities, such as hypertension, diabetes, and stroke, varied only modestly across omega-6 fatty acid tertiles. Similar patterns were observed when participants were stratified by plasma omega-3 fatty acid levels (Supplementary Table S3).

**Table 1:** Baseline characteristics by omega 6 fatty acids levels.

Variable	Total (*n* = 81,827)	Omega 6 fatty acids levels			*P* value
		Q1 (*n* = 27,292)	Q2 (*n* = 27,266)	Q3 (*n* = 27,269)	
**Age, years**	55.81 ± 8.27	55.34 ± 8.77	55.59 ± 8.20	56.51 ± 7.75	**< 0.0001**
Gender					
Female	44,871 (54.84)	12,149 (44.51)	15,323 (56.20)	17,399 (63.81)	**< 0.0001**
Male	36,956 (45.16)	15,143 (55.49)	11,943 (43.80)	9,870 (36.19)	
**BMI, kg/m** ^**3**^	27.51 ± 4.84	27.69 ± 5.13	27.36 ± 4.83	27.46 ± 4.54	**< 0.0001**
Ethnicity					
Missing	1,205 (1.47)	429 (1.57)	389 (1.43)	387 (1.42)	**< 0.0001**
Others	13,300 (16.25)	4,989 (18.28)	4,006 (14.69)	4,305 (15.79)	
White	67,322 (82.27)	21,874 (80.15)	22,871 (83.88)	22,577 (82.79)	
Education					
No degree/professional	27,407 (33.49)	8,962 (32.84)	9,247 (33.91)	9,198 (33.73)	**< 0.0001**
Degree/professional	38,375 (46.90)	12,888 (47.22)	12,958 (47.52)	12,529 (45.95)	
Missing	16,045 (19.61)	5,442 (19.94)	5,061 (18.56)	5,542 (20.32)	
**TDI**	−0.77 ± 3.34	−0.51 ± 3.48	−0.87 ± 3.29	−0.93 ± 3.24	**< 0.0001**
Household income, *£*/year					
< 18,000	17,341 (21.19)	6,056 (22.19)	5,416 (19.86)	5,869 (21.52)	**< 0.0001**
18,000 to 30,999	17,307 (21.15)	5,658 (20.73)	5,732 (21.02)	5,917 (21.70)	
31,000 to 51,999	17,060 (20.85)	5,624 (20.61)	5,842 (21.43)	5,594 (20.51)	
52,000 to 100,000	13,041 (15.94)	4,477 (16.40)	4,565 (16.74)	3,999 (14.67)	
>100,000	3,566 (4.36)	1,240 (4.54)	1,313 (4.82)	1,013 (3.71)	
Missing	13,512 (16.51)	4,237 (15.52)	4,398 (16.13)	4,877 (17.88)	
Smoking status					
Current	9,448 (11.55)	3,374 (12.36)	3,052 (11.19)	3,022 (11.08)	**< 0.0001**
Missing	663 (0.81)	253 (0.93)	190 (0.70)	220 (0.81)	
Never	44,303 (54.14)	14,364 (52.63)	14,856 (54.49)	15,083 (55.31)	
Previous	27,413 (33.50)	9,301 (34.08)	9,168 (33.62)	8,944 (32.80)	
Alcohol drinking status					
Current	72,167 (88.19)	23,821 (87.28)	24,328 (89.22)	24,018 (88.08)	**< 0.0001**
Missing	464 (0.57)	170 (0.62)	139 (0.51)	155 (0.57)	
Never	5,970 (7.30)	1,969 (7.21)	1,847 (6.77)	2,154 (7.90)	
Previous	3,226 (3.94)	1,332 (4.88)	952 (3.49)	942 (3.45)	
Physical activity level					
Low	22,779 (27.84)	7,855 (28.78)	7,534 (27.63)	7,390 (27.10)	**< 0.0001**
High	39,441 (48.20)	13,012 (47.68)	13,373 (49.05)	13,056 (47.88)	
Missing	19,607 (23.96)	6,425 (23.54)	6,359 (23.32)	6,823 (25.02)	
DM					
No	79,652 (97.34)	25,931 (95.01)	26,793 (98.27)	26,928 (98.75)	**< 0.0001**
Yes	2,175 (2.66)	1,361 (4.99)	473 (1.73)	341 (1.25)	
Stroke					
No	81,188 (99.22)	26,951 (98.75)	27,103 (99.40)	27,134 (99.50)	**< 0.0001**
Yes	639 (0.78)	341 (1.25)	163 (0.60)	135 (0.50)	
Hypertensive disease					
No	76,363 (93.32)	24,706 (90.52)	25,725 (94.35)	25,932 (95.10)	**< 0.0001**
Yes	5,464 (6.68)	2,586 (9.48)	1,541 (5.65)	1,337 (4.90)	
**AD PRS**	0.03 ± 1.00	−0.02 ± 0.99	0.03 ± 1.00	0.07 ± 1.01	**< 0.0001**
AD PRSQ					
Low	16,353 (19.98)	5,740 (21.03)	5,454 (20.00)	5,159 (18.92)	**< 0.0001**
Intermediate	49,100 (60.00)	16,466 (60.33)	16,451 (60.34)	16,183 (59.35)	
High	16,374 (20.01)	5,086 (18.64)	5,361 (19.66)	5,927 (21.74)	
**Dementia**	1,335 (1.63)	536 (1.96)	402 (1.47)	397 (1.46)	0.135

BMI, body mass index; TDI, Townsend deprivation index; DM, diabetes mellitus; PRS, polygenic risk score; AD, Alzheimer's disease. Continuous variables are presented as mean ± SD, and categorical variables as *n* (%). £: represents the British Pound Sterling (GBP).

### 3.2 Association of fatty acid levels with dementia risk

In fully adjusted models (Model 2), higher plasma omega-6 fatty acid concentrations were significantly associated with a lower risk of dementia (HR per 1 mmol/L increase = 0.85; 95% CI: 0.79–0.93; *p* < 0.001). When analyzed categorically, this inverse association persisted across omega-6 fatty acid tertiles (Q2 vs. Q1: HR = 0.84; 95% CI; 0.74–0.97; *p* = 0.01; Q3 vs. Q1: HR = 0.81; 95% CI: 0.70–0.92; *p* = 0.002), demonstrating a consistent graded relationship across all models.

In contrast, plasma omega-3 fatty acid concentrations were not independently associated with incident dementia after multivariable adjustment (per 1 mmol/L increase, HR = 0.97; 95% CI: 0.77–1.23; *p* = 0.81). Tertile analyses likewise indicated no significant associations (Q3 vs. Q1: HR = 1.00; 95% CI: 0.87–1.15; *p* = 1.00; *p*-trend = 0.98). Similarly the omega-6/omega-3 ratio showed no meaningful association with dementia risk in the fully adjusted model (per 1-unit increase, HR = 1.00; 95% CI: 0.98–1.01; *p* = 0.56) and tertile comparisons revealed no consistent trends (Q3 vs. Q1: HR = 0.89; 95% CI: 0.77–1.02; *p* = 0.10; *p*-trend = 0.10) (Table 2).

**Table 2:** Association of fatty acid levels and AD-PRS with dementia risk.

Character	Crude model		Model 1		Model 2	
	HR (95% CI)	*P*	HR (95% CI)	*P*	HR (95% CI)	*P*
**Omega 3 fatty acids mmol/l**	1.68 (1.36,2.09)	< 0.0001	0.89 (0.70,1.13)	0.33	0.97 (0.77,1.23)	0.81
Omega 3 fatty acids Q						
Q1	ref		ref		ref	
Q2	1.14 (1.00,1.31)	0.06	0.96 (0.83,1.10)	0.55	0.99 (0.86,1.13)	0.84
Q3	1.38 (1.21,1.57)	< 0.0001	0.95 (0.83,1.09)	0.44	1 (0.87,1.15)	1.00
*p* for trend		< 0.0001		0.45		0.98
**Omega 6 fatty acids mmol/l**	0.78 (0.72,0.84)	< 0.0001	0.82 (0.76,0.89)	< 0.0001	0.85 (0.79,0.93)	< 0.001
Omega 6 fatty acids Q						
Q1	ref		ref		ref	
Q2	0.73 (0.64,0.83)	< 0.0001	0.8 (0.70,0.91)	< 0.001	0.84 (0.74,0.97)	0.01
Q3	0.72 (0.63,0.82)	< 0.0001	0.76 (0.66,0.87)	< 0.0001	0.81 (0.70,0.92)	0.002
*p* for trend		< 0.0001		< 0.0001		0.002
**Omega 6/3 ratio**	0.95 (0.94,0.97)	< 0.0001	1 (0.99,1.01)	0.88	1 (0.98,1.01)	0.56
Omega 6/3 ratio Q						
Q1	ref		ref		ref	
Q2	0.77 (0.68,0.88)	< 0.0001	0.98 (0.86,1.11)	0.70	0.96 (0.85,1.09)	0.55
Q3	0.57 (0.49,0.65)	< 0.0001	0.91 (0.80,1.05)	0.21	0.89 (0.77,1.02)	0.10
*p* for trend		< 0.0001		0.22		0.1
**AD-PRS**	1.66 (1.59,1.74)	< 0.0001	1.7 (1.62,1.78)	< 0.0001	1.71 (1.63,1.79)	< 0.0001
AD-PRS Q						
Low	ref		ref		ref	
Intermediate	1.32 (1.11,1.58)	0.002	1.35 (1.13,1.61)	< 0.001	1.35 (1.13,1.61)	< 0.001
High	3.61 (3.02,4.31)	< 0.0001	3.8 (3.18,4.54)	< 0.0001	3.83 (3.21,4.58)	< 0.0001
*p* for trend		< 0.0001		< 0.0001		< 0.0001

BMI, body mass index; TDI, Townsend deprivation index; PRS, polygenic risk score; AD, Alzheimer's disease; HR, hazard ratios; CI, confidence intervals. Crude model was adjusted for no covariates. Model 1 was adjusted for age, gender, ethnicity, education, TDI; Model 2 was additionally adjusted for household income, BMI, alcohol drinking status, smoking status, physical activity level, hypertension disease, DM, stroke.

**Figure 2:**
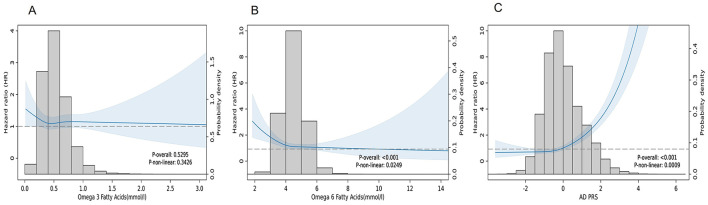
Linear and nonlinear association between fatty acids/AD-PRS and risk of dementia. **(A)** Omega-3 fatty acids; **(B)** Omega-6 fatty acids; **(C)** AD-PRS. BMI, body mass index; TDI, Townsend deprivation index; PRS, polygenic risk score; AD, Alzheimer's disease. Hazard ratios (HR) and 95% confidence intervals (CI) were estimated using Cox proportional hazard models with adjustment for age, gender, ethnicity, education, TDI, household income, BMI, alcohol drinking status, smoking status, physical activity level, hypertension disease, DM, stroke.

Restricted cubic spline (RCS) analyses demonstrated no significant linear or non-linear association for omega-3 (*p* for non-linear = 0.3426) (Figure 2A). By contrast, omega-6 exhibited a significant overall inverse association with dementia risk, with modest non-linear pattern (*p* for non-linear = 0.0249; reference = median value of 4.462 mmol/L) (Figure 2B). Dementia risk declined steeply up to the midpoint omega-6 concentrations and then plateaued at intermediate levels, with a slight upward trend at higher concentrations. The widening confidence intervals at the extremes suggest reduced data density in these ranges. Overall, omega-6 fatty acids exhibited a modest but robust protective association with dementia, whereas AD-PRS showed the strongest and most consistent risk gradient across all models.

### 3.3 Association of genetic risk score with dementia

Across three AD-PRS risk categories (low, intermediate, high), dementia events occurred in 155 participants (0.95%) in the low-risk group, 623 participants (1.27%) in the intermediate group, and 557 participants (3.40%) in the high-risk group, demonstrating a clear stepwise increase in risk. Genetic susceptibility, quantified by the AD-PRS, exhibited the strongest and most consistent association with dementia risk among all exposures examined. Each 1-SD increase in AD-PRS was significantly associated with a higher risk of dementia (HR = 1.71; 95% CI: 1.63–1.79; *p* < 0.0001). A clear dose-response relationship emerged across the three risk categories. Compared with individuals in the low-risk group, those in the intermediate group had a significantly increased risk of dementia (HR = 1.35; 95% CI: 1.13–1.61; *p* < 0.001), while those in the high-risk group exhibited a markedly elevated risk (HR = 3.83; 95% CI: 3.21–4.58; *p* < 0.0001; *p*-trend < 0.0001) (Table 2).

A monotonic association was also observed in restricted cubic spline analyses (Figure 2C).

### 3.4 Joint effects and interactions of fatty acids and genetic risk score on dementia

**Figure 3:**
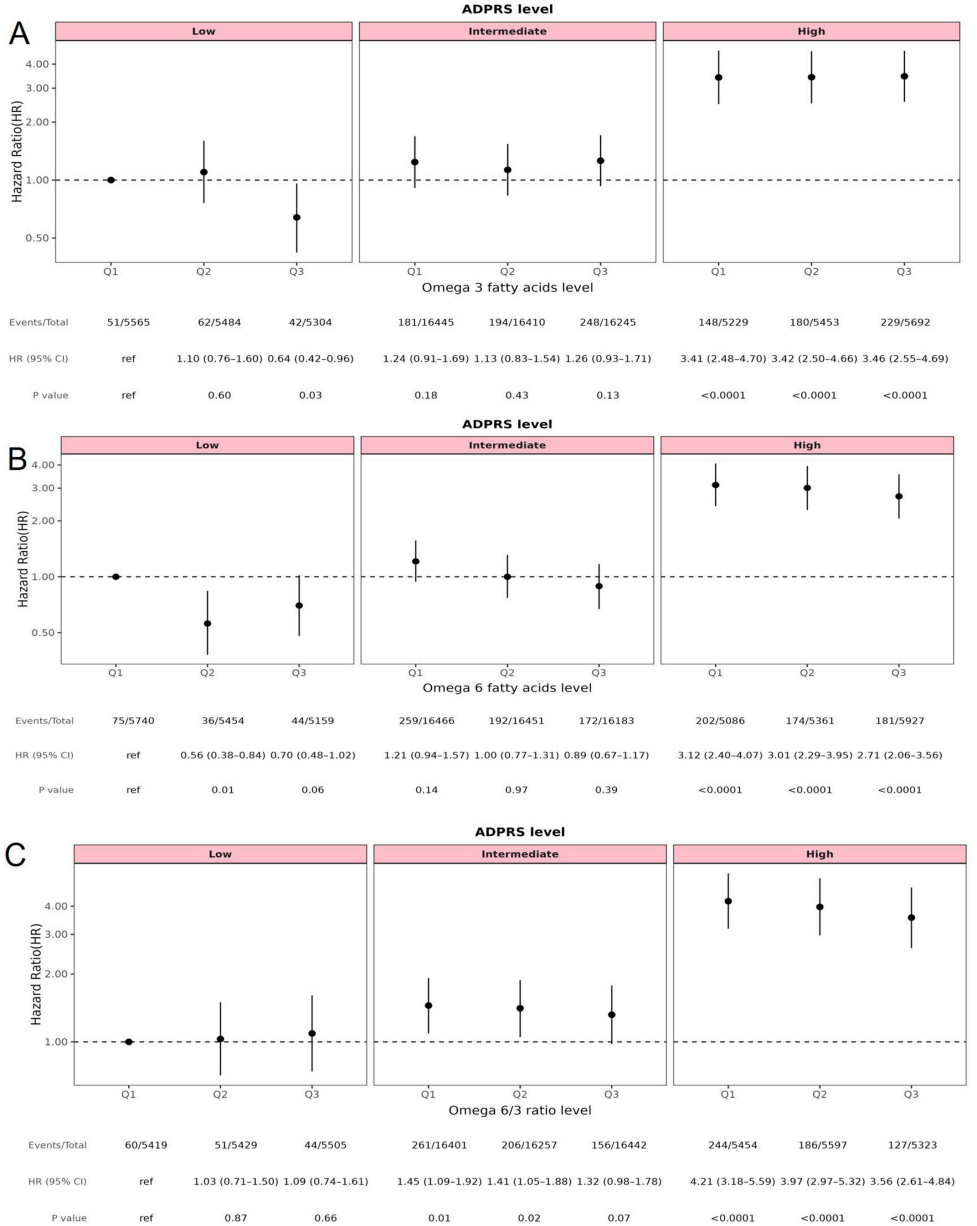
Joint effects of fatty acids and polygenic risk score on the risk of dementia. **(A)** Omega 3 fatty acids; **(B)** Omega 6 fatty acids; **(C)** Omega 6/3 ratio. BMI, body mass index; TDI, Townsend deprivation index; PRS, polygenic risk score; AD, Alzheimer's disease. AD PRS levels was categorized into low (the bottom quintile), intermediate (quintiles 2–4) and high (the top quintile) groups according to the distributions of PRS. Fatty acids level was categorized into Q1 (the bottom quintile)-Q3 (the top quintile) groups according to the distributions of fatty acids concentration. Hazard ratios (HR) and 95% confidence intervals (CI) were estimated using Cox proportional hazard models with adjustment for age, gender, ethnicity, education, TDI, household income, BMI, alcohol drinking status, smoking status, physical activity level, hypertension disease, DM, stroke.

Across combinations of omega fatty acid tertiles (Q1–Q3) and AD-PRS strata, the number of dementia events ranged from 51 to 75 in the low AD-PRS group, 181 to 261 in the intermediate group, and 148 to 244 in the high AD-PRS group, with approximately 5,000–6,500 participants in each category (Figure 3). Within the high AD-PRS stratum, dementia risk remained consistently more than threefold higher across all omega-3 categories (all *p* < 0.0001), indicating that omega-3 levels did not meaningfully offset elevated genetic susceptibility. In contrast, among individuals in the low AD-PRS stratum, higher omega-3 concentrations were associated with a significantly lower risk of dementia. Compared with participants in the low omega-3 category, those in the highest category had a HR of 0.64 (95% CI: 0.42–0.96; *p* = 0.03), suggesting a potential protective effect restricted to individuals with low genetic liability (Figure 3A).

Among participants with high AD-PRS, dementia risk was elevated across all omega-6 tertiles; however, the magnitude of risk decreased with increasing omega-6 levels, indicating a modest attenuation of genetic risk. For example, individuals in the highest omega-6 tertile (Q3-High) had an HR of 2.71 (95% CI: 2.06–3.56), compared with an HR of 3.12 (95% CI, 2.40–4.07) among those in the lowest tertile (Q1-High) (both *p* < 0.0001). Although absolute risk remained high, these findings suggest that higher omega-6 levels may partially mitigate the excess dementia risk associated with elevated AD-PRS (Figure 3B).

A similar pattern was observed for the omega-6/omega-3 ratio. In the high AD-PRS stratum, dementia risk remained substantially elevated across all ratio categories, but declined modestly with higher ratios [Q3-High: HR = 3.56 (95% CI: 2.61–4.84) vs. Q1-High: HR = 4.21 (95% CI: 3.18–5.59); *p* < 0.0001]. This attenuation again suggests that higher omega-6: omega-3 ratios may slightly reduce the genetic risk burden, although the magnitude of the effect was small (Figure 3C).

Overall, the joint analyses reinforce that genetic liability, as captured by AD-PRS, is the predominant determinant of dementia risk. While higher omega-3 levels among individuals with low genetic susceptibility and higher omega-6 or omega-6/omega-3 ratios among those with high genetic susceptibility were associated with modest risk reductions, these patterns were consistent with the primary analyses and did not substantially alter the dominant influence of genetic risk.

Additive interaction analyses revealed no statistically significant interactions between omega-3, omega-6, or the omega-6/omega-3 ratio and AD-PRS in relation to dementia risk. For omega-3 (Q3), a weak positive interaction signal was observed (RERI = 0.43, 95% CI: −0.35 to 1.20; AP = 0.12, 95% CI: −0.10 to 0.35; S = 1.21, 95% CI: 0.75 to 1.67), but the confidence intervals crossed the null, indicating no meaningful modification of genetic risk. For omega-6 (Q3), the interaction estimates were slightly negative (RERI = −0.16, 95% CI: −0.84 to 0.53; AP = −0.06, 95% CI: −0.32 to 0.20; S = 0.91, 95% CI: 0.56 to 1.27), suggesting a modest, but statistically non-significant, attenuation of the excess genetic risk among individuals with higher omega-6 levels (Supplementary Table S4). These findings are consistent with the joint analyses and suggest that omega-6 concentrations may partially buffer polygenic susceptibility, although the evidence remains inconclusive.

### 3.5 Subgroup analysis

**Figure 4:**
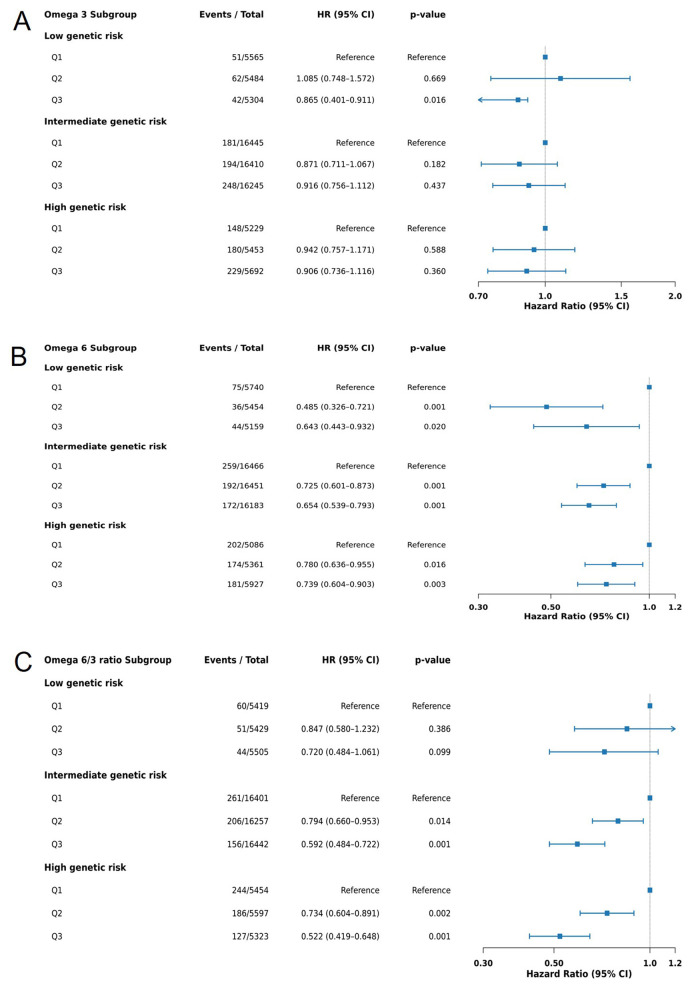
Subgroup analysis of fatty acid levels and dementia risk across different polygenic risk score categories. **(A)** Omega 3 fatty acids; **(B)** Omega 6 fatty acids; **(C)** Omega 6/3 ratio. BMI, body mass index; TDI, Townsend deprivation index; PRS, polygenic risk score; AD, Alzheimer's disease. AD PRS levels were categorized into low (the bottom quintile), intermediate (quintiles 2–4) and high (the top quintile) groups according to the distributions of PRS. Fatty acids level was categorized into Q1 (the bottom quintile)-Q3(the top quintile) groups according to the distributions of fatty acids concentration. Hazard ratios (HR) and 95% confidence intervals (CI) were estimated using Cox proportional hazard models with adjustment for age, gender, ethnicity, education, TDI, household income, BMI, alcohol drinking status, smoking status, physical activity level, hypertension disease, DM, stroke.

Plasma omega-3 concentrations demonstrated a consistent but non-significant trend toward lower dementia risk across predefined subgroups stratified by age, sex, lifestyle, and comorbidities (all p for interaction >0.05). However, within the low AD-PRS subgroup, higher omega-3 levels were significantly associated with a reduced risk of dementia (Q3 vs. Q1: HR = 0.60, 95% CI: 0.40–0.91, *p* = 0.016) (Figure 4A, Supplementary Table S5). Given the limited statistical power of subgroup analyses, these findings should be interpreted as exploratory.

In contrast, omega-6 fatty acids exhibited a robust and statistically significant inverse association with dementia risk across multiple subgroups. The protective effect was most pronounced among men, non-smokers, current alcohol drinkers, and participants without hypertension, DM, or stroke. Among men, the associations were particularly strong (Q3 vs. Q1: HR = 0.529, 95% CI: 0.431–0.648, *p* < 0.0001; Q2 vs. Q1: HR = 0.651, 95% CI: 0.545–0.778, *p* < 0.0001; p for interaction < 0.0001). Across all AD-PRS strata, higher omega-6 concentrations were consistently associated with lower dementia risk, with greater risk reduction observed at higher omega-6 levels. Although the association appeared somewhat attenuated among individuals with high genetic risk, these interactions were not statistically significant (Figure 4B, Supplementary Table S6).

The omega-6/omega-3 ratio showed a broadly similar pattern to omega-6 alone, with inverse associations across most subgroups, particularly among men and individuals with high AD-PRS, but without significant evidence of effect modification (Figure 4C, Supplementary Table S7).

Overall, AD-PRS emerged as the primary determinant of dementia risk. While higher omega-6 levels were consistently associated with reduced risk across both demographic and genetic subgroups, the protective associations of omega-3 were weaker and largely confined to individuals with low genetic liability. These findings suggest that the impact of fatty acid profiles on dementia risk may be partially dependent on underlying genetic liability.

### 3.6 Sensitivity analyses

Across multiple sensitivity analyses, the overall pattern of associations remained largely consistent with the primary findings. In analyses of the full cohort with available omega-3 and omega-6 data, both fatty acids initially appeared to be inversely associated with dementia risk (omega-3: HR = 0.80, 95% CI: 0.70–0.91, *p* = 0.001; omega-6: HR = 0.87, 95% CI: 0.84–0.91, *p* < 0.0001). However, after excluding individuals who developed dementia within the first three years of follow-up to minimize potential reverse causation, omega-3 was no longer significantly associated with dementia risk, whereas omega-6 retained a robust protective association (HR = 0.86, 95% CI: 0.79–0.94, *p* < 0.001). Similar results were observed in competing-risk analyses using Fine-Gray models, in which omega-3 again showed no significant association, and omega-6 remained protective (subdistribution HR = 1.00, 95% CI: 0.76–1.32, *p* = 0.98). Using age instead of follow-up time as the underlying time scale yielded comparable findings (HR = 0.88, 95% CI: 0.81–0.95, *p* = 0.001). Additional adjustment for correlated exposures and for AD-PRS did not materially alter the results. Omega-3 remained non-significant, whereas omega-6 continued to demonstrate a significant inverse association (HR = 0.82, 95% CI: 0.75–0.89, *p* < 0.0001). Excluding participants younger than 60 years at baseline produced similar results (HR = 0.84, 95% CI: 0.77–0.92, *p* < 0.001). Complete-case analyses that excluded individuals with missing covariates also supported the primary conclusions (HR = 0.83, 95% CI: 0.73–0.95, *p* = 0.01). When AD was examined as an alternative outcome, omega-3 showed no association (HR = 1.07, 95% CI: 0.76–1.51, *p* = 0.69), and omega-6 demonstrated a non-significant protective trend (HR = 0.91, 95% CI: 0.81–1.03, *p* = 0.14), likely reflecting limited statistical power. In contrast, AD-PRS remained a strong and consistent predictor of disease risk (Supplementary Tables S8–S15).

## 4 Discussion

### 4.1 Summary of findings

In this large prospective analysis of 81,827 UK Biobank participants, genetic susceptibility to AD, quantified using the AD-PRS, emerged as the dominant determinant of incident dementia. Each 1-SD increase in AD-PRS was associated with a 71% higher dementia risk. Beyond genetic liability, higher plasma concentrations of omega-6 fatty acids were independently associated with a lower risk of dementia, corresponding to an approximately 15% reduction per 1 mmol/L increase. In contrast, plasma omega-3 levels and the omega-6/omega-3 ratio were not significantly associated with dementia risk after multivariable adjustment. No statistically significant interactions were observed between genetic risk and any fatty acid biomarker. Although individuals with high genetic susceptibility exhibited slightly lower absolute dementia risk at higher omega-6 levels, this modifying effect was modest. Taken together, these findings indicate that genetic predisposition remains the primary driver of dementia risk, while plasma omega-6 levels confer an independent but comparatively smaller protective association.

### 4.2 Comparison with previous studies and interpretation

The inverse association between plasma omega-6 fatty acids and dementia risk observed in this study is consistent with several prospective investigations reporting that higher circulating omega-6 levels, or specific omega-6 components, are associated with slower cognitive decline and reduced dementia incidence (13, 14). These findings are biologically plausible, given the role of omega-6 fatty acids in preserving neuronal membrane integrity and modulating inflammatory pathways (27–29). However, the overall literature on omega-6 PUFAs and dementia remains mixed. Some ecological, cross-sectional, and case-control studies have linked higher dietary omega-6 intake or an elevated omega-6/omega-3 ratio to increased dementia risk (16, 30). Such inconsistencies likely reflect methodological differences, including reliance on self-reported dietary assessments, variation in population characteristics, and limitations inherent to non-prospective study designs (12, 18). By contrast, the present study leverages objectively measured plasma biomarkers within a large prospective cohort, thereby strengthening causal inference and supporting a protective association of omega-6 fatty acids.

In contrast to omega-6, plasma omega-3 fatty acids were not associated with dementia risk in fully adjusted models. This null finding aligns with the largely inconclusive results of long-term randomized controlled trials of omega-3 supplementation on cognitive outcomes (9, 10), despite contrasting with many observational studies that have reported protective associations (7, 8). The discrepancy between observational and interventional evidence underscores the susceptibility of nutritional epidemiology to residual confounding and reverse causation. Importantly, our analyses accounted for a wide range of sociodemographic, lifestyle, clinical, and genetic factors, including AD-PRS, suggesting that previously reported omega-3-dementia associations may be substantially attenuated when genetic susceptibility is rigorously controlled (31, 32). Similarly, we observed no association between the omega-6/omega-3 ratio and dementia risk, further highlighting the heterogeneity of findings across studies and the challenges of interpreting dietary ratios.

Joint anlyses provided limited evidence that higher omega-6 levels might modestly reduce absolute dementia risk even among individuals with high genetic susceptibility, although formal tests for interaction were not statistically significant. This pattern is consistent with the broader literature indicating that modifiable metabolic and lifestyle factors can partially mitigate genetically conferred dementia risk (21, 24). Nevertheless, the magnitude of this potential benefit was small compared with the strong and persistent influence of genetic predisposition. One speculative mechanism is that APOE genotype affects brain PUFA transport and utilization (15), while omega-6 fatty acids help maintain membrane integrity and modulate neuroinflammation (3, 15); higher omega-6 levels might therefore partially offset genetically driven inefficiencies, though this requires direct testing.

From a mechanistic perspective, total circulating omega-6 PUFA concentration represents a composite biomarker encompassing multiple fatty acid species with diverse biological functions. The observed association with dementia risk may therefore reflect not only the direct neurobiological effects of specific omega-6 derivatives but also broader links with overall diet quality, metabolic health, and systemic inflammatory status (15, 33). Future studies incorporating detailed lipidomic profiling to disentangle individual omega-6 species and their bioactive metabolites, alongside longitudinal designs, will be critical to clarifying the biological pathways underlying these associations (31, 34).

### 4.3 Strengths and limitations

This study possesses several important strengths. It draws on the large, prospective UK Biobank cohort, characterized by substantial sample size and extended follow-up, providing a robust framework for evaluating associations between circulating fatty acids and incident dementia while reducing the statistical instability commonly observed in smaller studies. Plasma omega-3, omega-6, and related lipid measures were quantified using standardized NMR metabolomics, enabling objective and reliable exposure assessment compared with self-reported dietary questionnaires, which are prone to measurement error. By incorporating the AD-PRS into a unified analytical model, this study was able to evaluate both the independent and joint effects of fatty acid metabolism and genetic susceptibility, an approach rarely undertaken in previous UK Biobank analyses. Importantly, we assessed interactions on both additive and multiplicative scales, with primary emphasis on the additive scale given its greater relevance for public health interpretation and causal inference. The inclusion of multiple prespecified sensitivity analyses further strengthened the robustness and internal validity of the findings.

Despite these advantages, several limitations should be acknowledged. First, plasma PUFA levels were measured only once at baseline, which may not fully reflect long-term or life-course exposure, particularly in the presence of potential intra-individual variability. Fatty acid levels can fluctuate with fasting status, recent dietary intake, and circadian rhythms: thus, a single measurement may only partially capture habitual metabolic profiles. Second, analyses were restricted to participants with both NMR metabolomics and genetic data, which may introduce selection bias and limit generalizability. Replication in larger samples, as AD-PRS availability continues to expand, will therefore be important. Third, although extensive covariate adjustment was undertaken, residual or unmeasured confounding cannot be ruled out. In particular, observed associations between fatty acids and dementia risk may partially reflect underlying differences in fish consumption, supplement use, overall diet quality, or socioeconomic status that are difficult to fully capture despite adjustment. Furthermore, although plasma fatty acids reflect integrated metabolic exposure, they may still be influenced by these upstream behavioral factors. In addition, plasma omega-6 levels may be correlated with broader lipid metabolic profiles. However, given the high correlation among NMR-derived lipid biomarkers, further adjustment for conventional lipid measures (e.g., LDL-C or HDL-C) may introduce overadjustment bias or multicollinearity. Similarly, because dietary patterns and supplement use are important determinants of circulating fatty acid levels, additional adjustment for these variables may also introduce overadjustment and may not necessarily improve confounding control. Therefore, residual confounding by detailed dietary and lipid-related factors cannot be entirely excluded. Fourth, dementia cases were ascertained using hospital admissions and death registry data, which may underidentify milder or non-hospitalized cases and thereby underestimate incidence; however, prior validation studies have demonstrated strong concordance between these sources and dementia diagnoses derived from primary care records (25). Fifth, despite the large cohort size and lengthy follow-up, the number of incident dementia events, particularly among the oldest participants, remains relatively limited. Sixth, as an observational study, causal inference cannot be established. Further research, including randomized controlled trials, longitudinal lipidomic trajectory analyses, and integrative multi-omics studies, will be required to elucidate the mechanistic pathways linking fatty acid metabolism to neurodegeneration. Finally, the predominance of participants of European ancestry limits the generalizability of our findings to more diverse populations.

## 5 Conclusion

In this large prospective analysis of the UK Biobank, higher plasma omega-6 concentrations were consistently associated with lower dementia risk, whereas neither omega-3 levels nor the omega-6/omega-3 ratio showed significant associations. Although higher omega-6 levels were associated with slightly lower absolute risk among individuals with elevated genetic susceptibility to AD (for example, the hazard ratio dropped from about 3.1 to 2.7), genetic predisposition remained the dominant risk factor, and this modest effect does not imply that omega-6 supplementation can counteract high genetic risk. These findings suggest that omega-6 fatty acids may influence neurodegenerative processes through pathways partially independent of genetic liability. Further randomized trials, large-scale cohort studies, and mechanistic investigations will be essential to confirm these associations and clarify the underlying biological mechanisms.

## Data Availability

The original contributions presented in the study are included in the article/Supplementary material, further inquiries can be directed to the corresponding author.
