# Supplementary material for: Higher plasma omega-6 fatty acids are associated with lower dementia risk: evidence from NMR metabolomics and Alzheimer's disease polygenic risk in the UK Biobank
Source: Front Public Health. 2026 May 14;14:1816467. doi: 10.3389/fpubh.2026.1816467 (PMC13216053; doi:10.3389/fpubh.2026.1816467)
Supplement: Supplementary file 1 [file Table_1.docx]

# Title：

Higher Plasma Omega-6 Fatty Acids Are Associated With Lower Dementia Risk: Evidence From NMR Metabolomics and AD Polygenic Risk in the UK Biobank

## **Supplementary Materials Content**

Supplementary Table S1. Field ID in UK biobank of variables in this study.

Supplementary Table S2. ICD codes for variables in this study.

Supplementary Table S3. Baseline characteristics of participants by omega 3 fatty acids

Supplementary Table S4. Measures of additive interaction between PRS and omega-3/omega-6 fatty acids on dementia risk

Supplementary Table S5. Subgroup analyses for the associations between omega 3 fatty acids and the risk of dementia

Supplementary Table S6. Subgroup analyses for the associations between omega 6fatty acids and the risk of dementia

Supplementary Table S7. Subgroup analyses for the associations between omega 6/3 fatty acids ratio and the risk of dementia

Supplementary Table S8. Sensitivity analysis 1 of the association between omega-3 and omega-6 fatty acids and dementia risk in the cohort without AD PRS data

Supplementary Table S9. Sensitive analysis 2: the association between omega-3 and omega-6 fatty acids and dementia risk, excluding cases diagnosed within 3 years

Supplementary Table S10. Sensitive analysis 3: the association between omega-3 and omega-6 fatty acids and dementia risk using competing risk cox model

Supplementary Table S11. Sensitive analysis 4: the association between omega-3 and omega-6 fatty acids and dementia risk using Age replace the follow-up time in cox proportional hazards analysis

Supplementary Table S12. Sensitive analysis 5: the association between omega-3 and omega-6 fatty acids and dementia risk with mutual adjustment of omega-3, omega-6, and AD PRS in cox regression analysis

Supplementary Table S13. Sensitive analysis 6: the association between omega-3 and omega-6 fatty acids and dementia risk with aged more than 60 years old

Supplementary Table S14. Sensitive analysis 7: the association between omega-3 and omega-6 fatty acids and dementia risk with excluding all missing data

Supplementary Table S15. Sensitive analysis 8: the association between omega-3 and omega-6 fatty acids and Alzheimer’s Disease risk

Supplementary Table S1 Field ID in UK biobank of variables in this study.

| **Variables** | **Fields ID in the UK biobank** |
| --- | --- |
| Age | 21003 |
| Gender | 31 |
| Ethnicity | 21000 |
| Education | 6138 |
| TDI | 189 |
| Household income | 738 |
| Smoking status | 20116 |
| Alcohol drinking status | 20117 |
| Physical activity levels | 884, 894, 904,914 |
| BMI | 21001 |
| AD-PRS | 26207 |
| Omega-3 Fatty Acids | 23444 |
| Omega-6 Fatty Acids | 23446 |
| Omega-6 Fatty Acids to Omega-3 Fatty Acids ratio | 23459 |

Supplementary Table S2. ICD codes for variables in this study.

| **Disease** | **ICD-9 code** | **ICD-10 code** |
| --- | --- | --- |
| Dementia | 290.2, 290.3, 290.4, 291.2, 294.1, 331.0, 331.1, 331.2, 331.5 | F00, F01, F02, F03, F05.1, F10.6, G30, G31.0, G31.1, G31.8, I67.3, A81.0 |
| Alzheimer’s disease | 3310 | G30, F00 |
| Hypertensive disease | 401, 402, 403, 404, 405 | I10, I11, I12, I13, I14, I15 |
| Diabetes mellitus | 250, 250.0, 250.1, 250.2, 250.3, 250.4, 250.5, 250.6, 250.7, 250.8, 250.9 | E10, E11, E12, E13, E14 |
| Stroke | 430, 431, 432, 433, 434, 435, 436, 437, 438 | I60, I61, I62, I63, I64, I65, I66, I67, I68, I69 |

ICD-9,International Classification of Diseases, 9th Revision;

ICD-10,International Classification of Diseases, 10th Revision.

Supplementary Table S3 Baseline characteristics of participants by omega 3 fatty acids levels.

| **Variable** | **Total (n=81827)** | Omega 3 fatty acids levels | | | **P value** |
| --- | --- | --- | --- | --- | --- |
|  |  | **Q1 (n=27239)** | **Q2 (n=27347)** | **Q3 (n=27241)** |  |
| **Age, years** | 55.81 ± 8.27 | 53.88 ± 8.48 | 55.77 ± 8.20 | 57.79 ± 7.63 | **<0.0001** |
| **Gender** |  |  |  |  | **<0.0001** |
| Female | 44871(54.84) | 12937(47.49) | 14869(54.37) | 17065(62.64) |  |
| Male | 36956(45.16) | 14302(52.51) | 12478(45.63) | 10176(37.36) |  |
| **BMI, kg/m^3^** | 27.51 ± 4.84 | 27.38 ± 5.14 | 27.70 ± 4.86 | 27.44 ± 4.51 | **<0.0001** |
| **Ethnicity** |  |  |  |  | **<0.0001** |
| Missing | 1205( 1.47) | 447( 1.64) | 388( 1.42) | 370( 1.36) |  |
| Others | 13300(16.25) | 4845(17.79) | 4288(15.68) | 4167(15.30) |  |
| White | 67322(82.27) | 21947(80.57) | 22671(82.90) | 22704(83.34) |  |
| **Education** |  |  |  |  | **<0.0001** |
| No degree/professional | 27407(33.49) | 9624(35.33) | 9166(33.52) | 8617(31.63) |  |
| Degree/professional | 38375(46.90) | 12247(44.96) | 12805(46.82) | 13323(48.91) |  |
| Missing | 16045(19.61) | 5368(19.71) | 5376(19.66) | 5301(19.46) |  |
| **TDI** | -0.77 ± 3.34 | -0.47 ± 3.39 | -0.81 ± 3.34 | -1.03 ± 3.28 | **<0.0001** |
| **Household income, *£*/year** |  |  |  |  | **<0.0001** |
| 18,000 to 30,999 | 17307(21.15) | 5702(20.93) | 5684(20.78) | 5921(21.74) |  |
| 31,000 to 51,999 | 17060(20.85) | 5903(21.67) | 5771(21.10) | 5386(19.77) |  |
| 52,000 to 100,000 | 13041(15.94) | 4409(16.19) | 4528(16.56) | 4104(15.07) |  |
| Greater than 100,000 | 3566( 4.36) | 1056( 3.88) | 1219( 4.46) | 1291( 4.74) |  |
| Less than 18,000 | 17341(21.19) | 5985(21.97) | 5679(20.77) | 5677(20.84) |  |
| Missing | 13512(16.51) | 4184(15.36) | 4466(16.33) | 4862(17.85) |  |
| **Smoking status** |  |  |  |  | **<0.0001** |
| Current | 9448(11.55) | 4127(15.15) | 2999(10.97) | 2322( 8.52) |  |
| Missing | 663( 0.81) | 233( 0.86) | 216( 0.79) | 214( 0.79) |  |
| Never | 44303(54.14) | 14635(53.73) | 14705(53.77) | 14963(54.93) |  |
| Previous | 27413(33.50) | 8244(30.27) | 9427(34.47) | 9742(35.76) |  |
| **Alcohol drinking status** |  |  |  |  | **<0.0001** |
| Current | 72167(88.19) | 23306(85.56) | 24205(88.51) | 24656(90.51) |  |
| Missing | 464( 0.57) | 177( 0.65) | 137( 0.50) | 150( 0.55) |  |
| Never | 5970( 7.30) | 2448( 8.99) | 1944( 7.11) | 1578( 5.79) |  |
| Previous | 3226( 3.94) | 1308( 4.80) | 1061( 3.88) | 857( 3.15) |  |
| **Physical activity level** |  |  |  |  | **<0.01** |
| Low | 22779(27.84) | 7620(27.97) | 7690(28.12) | 7469(27.42) |  |
| High | 39441(48.20) | 12968(47.61) | 13098(47.90) | 13375(49.10) |  |
| Missing | 19607(23.96) | 6651(24.42) | 6559(23.98) | 6397(23.48) |  |
| **DM** |  |  |  |  | **<0.0001** |
| No | 79652(97.34) | 26411(96.96) | 26574(97.17) | 26667(97.89) |  |
| Yes | 2175( 2.66) | 828( 3.04) | 773( 2.83) | 574( 2.11) |  |
| **Stroke** |  |  |  |  | 0.10 |
| No | 81188(99.22) | 27012(99.17) | 27159(99.31) | 27017(99.18) |  |
| Yes | 639( 0.78) | 227( 0.83) | 188( 0.69) | 224( 0.82) |  |
| **Hypertensive disease** |  |  |  |  | **0.02** |
| No | 76363(93.32) | 25514(93.67) | 25476(93.16) | 25373(93.14) |  |
| Yes | 5464( 6.68) | 1725( 6.33) | 1871( 6.84) | 1868( 6.86) |  |
| **AD PRS** | 0.03 ± 1.00 | 0.01 ± 0.99 | 0.03 ± 1.00 | 0.05 ± 1.01 | **<0.0001** |
| **AD PRSQ** |  |  |  |  | **<0.0001** |
| Low | 16353(19.98) | 5565(20.43) | 5484(20.05) | 5304(19.47) |  |
| Intermediate | 49100(60.00) | 16445(60.37) | 16410(60.01) | 16245(59.63) |  |
| High | 16374(20.01) | 5229(19.20) | 5453(19.94) | 5692(20.89) |  |

Abbreviations: BMI, body mass index; TDI, Townsend deprivation index; DM, diabetes mellitus; PRS, polygenic risk score; AD, Alzheimer's disease. Continuous variables are presented as mean ± SD, and categorical variables as n (%). £: represents the British Pound Sterling (GBP).

Supplementary Table S4. Measures of additive interaction between PRS and omega-3/omega-6 fatty acids on dementia risk

| Variates | PRS high | | |
| --- | --- | --- | --- |
| Fatty acid | RERI (95% CI) | AP (95% CI) | S (95% CI) |
| Omega-3 Q3 | 0.43 (−0.35~1.20) | 0.12 (−0.10~0.35) | 1.21 (0.75~1.67) |
| Omega-6 Q3 | −0.16 (−0.84~0.53) | −0.06 (−0.32~0.20) | 0.91 (0.56~1.27) |

Abbreviations: BMI, body mass index; TDI, Townsend deprivation index; PRS, polygenic risk score; AD, Alzheimer's disease; HR, hazard ratios; CI, confidence intervals; PRS, polygenic risk score; RERI, relative excess risk due to interaction; AP, attributable proportion due to interaction; S, synergy index.

Adjusted for age, gender, ethnicity, education, TDI, household income, BMI, alcohol drinking status, smoking status, physical activity level, hypertension disease, DM, stroke.

Note: To estimate RERI, AP, and S, individuals with low PRS and the lowest tertile (Q1) of omega-3 or omega-6 levels were set as the reference group, and the results represent the comparison of high PRS and the highest tertile (Q3) of omega-3 or omega-6 vs. the reference group.

Supplementary Table S5. Subgroup analyses for the associations between omega 3 fatty acids and the risk of dementia

| Character | Q1 | Q2  HR(95%CI) | P | Q3  HR(95%CI) | P | P for trend | P for interaction |
| --- | --- | --- | --- | --- | --- | --- | --- |
| Gender |  |  |  |  |  |  | 0.321 |
| Female | ref | 1.039(0.834,1.295) | 0.733 | 1.085(0.883,1.332) | 0.439 | 0.418 |  |
| Male | ref | 0.933(0.779,1.117) | 0.449 | 0.863(0.713,1.044) | 0.129 | 0.129 |  |
| Smoking status |  |  |  |  |  |  | 0.748 |
| Current | ref | 0.714(0.475, 1.075) | 0.106 | 0.737(0.486, 1.117) | 0.151 | 0.122 |  |
| Missing | ref | 1.119(0.396, 3.159) | 0.833 | 1.138(0.398, 3.251) | 0.809 | 0.81 |  |
| Never | ref | 0.956(0.779,1.174) | 0.667 | 0.960(0.791,1.166) | 0.682 | 0.71 |  |
| Previous | ref | 1.001(0.806,1.243) | 0.994 | 0.922(0.745,1.142) | 0.457 | 0.424 |  |
| Alcohol drinking status |  |  |  |  |  |  | 0.64 |
| Current | ref | 0.971(0.835,1.130) | 0.705 | 0.908(0.784,1.052) | 0.199 | 0.183 |  |
| Missing | ref | 0.505(0.125, 2.044) | 0.338 | 0.525(0.129, 2.129) | 0.367 | 0.32 |  |
| Never | ref | 1.010(0.622,1.638) | 0.969 | 1.198(0.758,1.895) | 0.439 | 0.432 |  |
| Previous | ref | 0.662(0.393,1.116) | 0.121 | 0.850(0.523,1.380) | 0.510 | 0.478 |  |
| Hypertensive disease |  |  |  |  |  |  | 0.844 |
| no | ref | 0.943(0.815,1.091) | 0.428 | 0.916(0.796,1.055) | 0.224 | 0.232 |  |
| yes | ref | 0.893(0.587, 1.357) | 0.595 | 0.801(0.527, 1.219) | 0.300 | 0.301 |  |
| DM |  |  |  |  |  |  | 0.086 |
| no | ref | 0.978(0.847,1.129) | 0.757 | 0.921(0.801,1.059) | 0.249 | 0.233 |  |
| yes | ref | 0.631(0.379, 1.052) | 0.077 | 1.066(0.668, 1.701) | 0.788 | 0.887 |  |
| Stroke |  |  |  |  |  |  | 0.731 |
| no | ref | 0.946(0.823,1.088) | 0.435 | 0.908(0.793,1.039) | 0.160 | 0.162 |  |
| yes | ref | 0.744(0.295, 1.878) | 0.532 | 0.969(0.416, 2.259) | 0.942 | 0.957 |  |
| AD PRSQ |  |  |  |  |  |  | 0.052 |
| Low | ref | 1.085(0.748,1.572) | 0.669 | 0.604(0.401,0.911) | 0.016 | 0.014 |  |
| Intermediate | ref | 0.871(0.711,1.067) | 0.182 | 0.916(0.756,1.112) | 0.376 | 0.437 |  |
| High | ref | 0.942(0.757,1.171) | 0.588 | 0.906(0.736,1.116) | 0.355 | 0.36 |  |

Abbreviations: BMI, body mass index; TDI, Townsend deprivation index; PRS, polygenic risk score; AD, Alzheimer's disease; HR, hazard ratios; CI, confidence intervals.

HR and 95% CI were estimated using Cox proportional hazard models with adjustment for age, gender, ethnicity, education, TDI, household income, BMI, alcohol drinking status, smoking status, physical activity level, hypertension disease, DM, stroke, except the stratification variable.

Supplementary Table S6. Subgroup analyses for the associations between omega 6 fatty acids and the risk of dementia

| Character | Q1 | Q2  HR(95%CI) | P | Q3  HR(95%CI) | P | P for trend | P for interaction |
| --- | --- | --- | --- | --- | --- | --- | --- |
| Gender |  |  |  |  |  |  | < 0.0001 |
| Female | ref | 0.896(0.737,1.090) | 0.271 | 1.004(0.834,1.209) | 0.966 | 0.854 |  |
| Male | ref | 0.651(0.545,0.778) | <0.0001 | 0.529(0.431,0.648) | <0.0001 | <0.0001 |  |
| Smoking status |  |  |  |  |  |  | 0.249 |
| Current | ref | 0.833(0.559,1.241) | 0.368 | 0.694(0.456,1.057) | 0.089 | 0.086 |  |
| Missing | ref | 0.325(0.092,1.152) | 0.082 | 0.814(0.332,1.998) | 0.654 | 0.55 |  |
| Never | ref | 0.827(0.687,0.994) | 0.044 | 0.731(0.605,0.885) | 0.001 | 0.001 |  |
| Previous | ref | 0.618(0.502,0.762) | <0.0001 | 0.719(0.588,0.879) | 0.001 | <0.001 |  |
| Alcohol drinking status |  |  |  |  |  |  | 0.34 |
| Current | ref | 0.762(0.662,0.877) | <0.001 | 0.746(0.648,0.860) | <0.0001 | <0.0001 |  |
| Missing | ref | 0.273(0.059,1.264) | 0.097 | 0.229(0.049,1.059) | 0.059 | 0.034 |  |
| Never | ref | 0.491(0.299,0.806) | 0.005 | 0.657(0.427,1.010) | 0.056 | 0.048 |  |
| Previous | ref | 0.844(0.520,1.369) | 0.492 | 0.691(0.409,1.167) | 0.167 | 0.162 |  |
| Hypertensive disease |  |  |  |  |  |  | 0.449 |
| no | ref | 0.762(0.664,0.873) | <0.0001 | 0.752(0.656,0.863) | <0.0001 | <0.0001 |  |
| yes | ref | 0.588(0.384,0.902) | 0.015 | 0.626(0.401,0.976) | 0.039 | 0.014 |  |
| DM |  |  |  |  |  |  | 0.789 |
| no | ref | 0.761(0.665,0.871) | <0.0001 | 0.762(0.666,0.872) | <0.0001 | <0.0001 |  |
| yes | ref | 0.875(0.529,1.447) | 0.603 | 0.886(0.488,1.610) | 0.692 | 0.594 |  |
| Stroke |  |  |  |  |  |  | 0.36 |
| no | ref | 0.732(0.642,0.835) | <0.0001 | 0.735(0.645,0.838) | <0.0001 | <0.0001 |  |
| yes | ref | 0.940(0.425,2.078) | 0.878 | 0.388(0.115,1.313) | 0.128 | 0.157 |  |
| AD PRSQ |  |  |  |  |  |  | 0.269 |
| Low | ref | 0.485(0.326,0.721) | <0.001 | 0.643(0.443,0.932) | 0.020 | 0.009 |  |
| Intermediate | ref | 0.725(0.601,0.873) | <0.001 | 0.654(0.539,0.793) | <0.0001 | <0.0001 |  |
| High | ref | 0.780(0.636,0.955) | 0.016 | 0.739(0.604,0.903) | 0.003 | 0.003 |  |

Abbreviations: BMI, body mass index; TDI, Townsend deprivation index; PRS, polygenic risk score; AD, Alzheimer's disease; HR, hazard ratios; CI, confidence intervals.

HR and 95% CI were estimated using Cox proportional hazard models with adjustment for age, gender, ethnicity, education, TDI, household income, BMI, alcohol drinking status, smoking status, physical activity level, hypertension disease, DM, stroke, except the stratification variable.

Supplementary Table S7. Subgroup analyses for the associations between omega 6/3 fatty acids ratio and the risk of dementia

| Character | Q1 | Q2  HR(95%CI) | P | Q3  HR(95%CI) | P | P for trend | P for interaction |
| --- | --- | --- | --- | --- | --- | --- | --- |
| Gender |  |  |  |  |  |  | 0.01 |
| Female | ref | 0.680(0.570,0.808) | <0.0001 | 0.460(0.375,0.562) | <0.0001 | <0.0001 |  |
| Male | ref | 0.897(0.747,1.078) | 0.247 | 0.686(0.566,0.831) | <0.001 | <0.001 |  |
| Smoking status |  |  |  |  |  |  | 0.142 |
| Current | ref | 0.908(0.587,1.415) | 0.667 | 0.750(0.492,1.155) | 0.185 | 0.171 |  |
| Missing | ref | 0.963(0.388,2.393) | 0.935 | 0.301(0.067,1.001) | 0.072 | 0.083 |  |
| Never | ref | 0.683(0.569,0.818) | <0.0001 | 0.494(0.404,0.602) | <0.0001 | <0.0001 |  |
| Previous | ref | 0.891(0.732,1.083) | 0.248 | 0.689(0.551,0.858) | <0.001 | 0.001 |  |
| Alcohol drinking status |  |  |  |  |  |  | 0.078 |
| Current | ref | 0.790(0.689,0.905) | <0.001 | 0.586(0.503,0.680) | <0.0001 | <0.0001 |  |
| Missing | ref | 1.269(0.374,4.492) | 0.699 | 0.356(0.051,1.680) | 0.222 | 0.252 |  |
| Never | ref | 0.817(0.527,1.264) | 0.364 | 0.352(0.211,0.573) | <0.0001 | <0.0001 |  |
| Previous | ref | 0.494(0.282,0.841) | 0.011 | 0.624(0.384,1.009) | 0.055 | 0.061 |  |
| Hypertensive disease |  |  |  |  |  |  | 0.138 |
| no | ref | 0.756(0.662,0.863) | <0.0001 | 0.561(0.485,0.647) | <0.0001 | <0.0001 |  |
| yes | ref | 1.092(0.736,1.616) | 0.659 | 0.821(0.514,1.286) | 0.398 | 0.48 |  |
| DM |  |  |  |  |  |  | 0.888 |
| no | ref | 0.775(0.680,0.883) | <0.001 | 0.576(0.500,0.664) | <0.0001 | <0.0001 |  |
| yes | ref | 0.840(0.527,1.333) | 0.460 | 0.658(0.374,1.121) | 0.133 | 0.13 |  |
| Stroke |  |  |  |  |  |  | 0.944 |
| no | ref | 0.784(0.690,0.890) | <0.001 | 0.575(0.500,0.661) | <0.0001 | <0.0001 |  |
| yes | ref | 0.719(0.296,1.651) | 0.445 | 0.629(0.236,1.526) | 0.323 | 0.296 |  |
| AD PRSQ |  |  |  |  |  |  | 0.705 |
| Low | ref | 0.847(0.580,1.232) | 0.386 | 0.720(0.484,1.061) | 0.099 | 0.098 |  |
| Intermediate | ref | 0.794(0.660,0.953) | 0.014 | 0.592(0.484,0.722) | <0.0001 | <0.0001 |  |
| High | ref | 0.734(0.604,0.891) | 0.002 | 0.522(0.419,0.648) | <0.0001 | <0.0001 |  |

Abbreviations: BMI, body mass index; TDI, Townsend deprivation index; PRS, polygenic risk score; AD, Alzheimer's disease; HR, hazard ratios; CI, confidence intervals.

HR and 95% CI were estimated using Cox proportional hazard models with adjustment for age, gender, ethnicity, education, TDI, household income, BMI, alcohol drinking status, smoking status, physical activity level, hypertension disease, DM, stroke, except the stratification variable.

Supplementary Table S8. Sensitivity analysis 1 of the association between omega-3 and omega-6 fatty acids and dementia risk in the cohort without AD PRS data.(Event=4791, N=274702)

| **Character** | **Crude model** | | **Model 1** | | **Model 2** | |
| --- | --- | --- | --- | --- | --- | --- |
|  | HR(95%CI) | P | HR(95%CI) | P | HR(95%CI) | P |
| **Omega 3 Fatty Acids mmol/l** | 1.34(1.18,1.51) | <0.0001 | 0.72(0.63,0.82) | <0.0001 | 0.8(0.70,0.91) | 0.001 |
| **Omega 3 Fatty Acids Q** |  |  |  |  |  |  |
| Q1 | ref |  | ref |  | ref |  |
| Q2 | 1.03(0.96,1.10) | 0.47 | 0.85(0.79,0.91) | <0.0001 | 0.88(0.82, 0.95) | <0.001 |
| Q3 | 1.12(1.04,1.20) | 0.002 | 0.79(0.73,0.85) | <0.0001 | 0.84(0.78, 0.90) | <0.0001 |
| p for trend |  | 0.002 |  | <0.0001 |  | <0.0001 |
| **Omega 6 Fatty Acids mmol/l** | 0.77(0.74,0.81) | <0.0001 | 0.83(0.80,0.87) | <0.0001 | 0.87(0.84, 0.91) | <0.0001 |
| **Omega 6 Fatty Acids Q** |  |  |  |  |  |  |
| Q1 | ref |  | ref |  | ref |  |
| Q2 | 0.7(0.65,0.75) | <0.0001 | 0.78(0.73,0.84) | <0.0001 | 0.83(0.77, 0.89) | <0.0001 |
| Q3 | 0.7(0.66,0.75) | <0.0001 | 0.76(0.71,0.82) | <0.0001 | 0.82(0.76, 0.88) | <0.0001 |
| p for trend |  | <0.0001 |  | <0.0001 |  | <0.0001 |

Abbreviations: BMI, body mass index; TDI, Townsend deprivation index; PRS, polygenic risk score; AD, Alzheimer's disease; HR, hazard ratios; CI, confidence intervals.

Crude model was adjusted for no covariates. Model 1 was adjusted for age, gender, ethnicity, education, TDI; Model 2 was additionally adjusted for household income, BMI, alcohol drinking status, smoking status, physical activity level, hypertension disease, DM, stroke.

Supplementary Table S9. Sensitive analysis 2: the association between omega-3 and omega-6 fatty acids and dementia risk, excluding cases diagnosed within 3 years(Event=1278,N=81020)

| **Character** | **Crude model** | | **Model 1** | | **Model 2** | |
| --- | --- | --- | --- | --- | --- | --- |
|  | HR(95%CI) | P | HR(95%CI) | P | HR(95%CI) | P |
| **Omega 3 Fatty Acids mmol/l** | 1.78(1.43,2.21) | <0.0001 | 0.93(0.73,1.19) | 0.58 | 1.02(0.80,1.29) | 0.90 |
| **Omega 3 Fatty Acids Q** |  |  |  |  |  |  |
| Q1 | ref |  | ref |  | ref |  |
| Q2 | 1.19(1.04,1.37) | 0.01 | 1(0.86,1.15) | 0.96 | 1.02(0.89,1.18) | 0.75 |
| Q3 | 1.42(1.24,1.63) | <0.0001 | 0.97(0.85,1.12) | 0.70 | 1.02(0.89,1.18) | 0.74 |
| p for trend |  | <0.0001 |  | 0.69 |  | 0.75 |
| **Omega 6 Fatty Acids mmol/l** | 0.79(0.73,0.86) | <0.0001 | 0.83(0.77,0.90) | <0.0001 | 0.86(0.79,0.94) | <0.001 |
| **Omega 6 Fatty Acids Q** |  |  |  |  |  |  |
| Q1 | ref |  | ref |  | ref |  |
| Q2 | 0.73(0.64,0.84) | <0.0001 | 0.8(0.70,0.92) | 0.001 | 0.84(0.74,0.97) | 0.01 |
| Q3 | 0.73(0.64,0.84) | <0.0001 | 0.77(0.67,0.88) | <0.001 | 0.81(0.70,0.93) | 0.003 |
| p for trend |  | <0.0001 |  | <0.001 |  | 0.003 |
| **Omega 6/3 ratio** | 0.95(0.93,0.96) | <0.0001 | 1(0.98,1.01) | 0.62 | 0.99(0.98,1.01) | 0.35 |
| **Omega 6/3 ratio Q** |  |  |  |  |  |  |
| Q1 | ref |  | ref |  | ref |  |
| Q2 | 0.77(0.68,0.88) | <0.0001 | 0.98(0.86,1.11) | 0.75 | 0.97(0.85,1.10) | 0.60 |
| Q3 | 0.55(0.48,0.63) | <0.0001 | 0.9(0.78,1.03) | 0.13 | 0.87(0.75,1.00) | 0.06 |
| p for trend |  | <0.0001 |  | 0.15 |  | 0.07 |

Abbreviations: BMI, body mass index; TDI, Townsend deprivation index; PRS, polygenic risk score; AD, Alzheimer's disease; HR, hazard ratios; CI, confidence intervals.

Crude model was adjusted for no covariates. Model 1 was adjusted for age, gender, ethnicity, education, TDI; Model 2 was additionally adjusted for household income, BMI, alcohol drinking status, smoking status, physical activity level, hypertension disease, DM, stroke.

Supplementary Table S10. Sensitive analysis 3: the association between omega-3 and omega-6 fatty acids and dementia risk using competing risk cox model (N=81727)

| Variable | SHR (95% CI) | p-value |
| --- | --- | --- |
| **Omega 3 Fatty Acids mmol/l** | 1.00 (0.76–1.32) | 0.980 |
| **Omega 3 Fatty Acids Q** |  |  |
| Q1 | ref | ref |
| Q2 | 0.98 (0.83–1.15) | 0.790 |
| Q3 | 1.02 (0.87–1.19) | 0.820 |
| **Omega 6 Fatty Acids mmol/l** | 0.87 (0.78–0.95) | 0.004 |
| **Omega 6 Fatty Acids Q** |  |  |
| Q1 | ref | ref |
| Q2 | 0.85 (0.73–0.99) | 0.039 |
| Q3 | 0.79 (0.67–0.92) | 0.003 |

Abbreviations: BMI, body mass index; TDI, Townsend deprivation index; PRS, polygenic risk score; AD, Alzheimer's disease; HR, hazard ratios; CI, confidence intervals; SHR, subdistribution hazard ratio.

Crude model was adjusted for no covariates. Model 1 was adjusted for age, gender, ethnicity, education, TDI; Model 2 was additionally adjusted for household income, BMI, alcohol drinking status, smoking status, physical activity level, hypertension disease, DM, stroke.

Supplementary Table S11. Sensitive analysis 4:the association between omega-3 and omega-6 fatty acids and dementia risk using Age replace the follow-up time in cox proportional hazards analysis (N=81827)

| **Character** | **Crude model** | | **Model 1** | | **Model 2** | |
| --- | --- | --- | --- | --- | --- | --- |
|  | HR(95%CI) | P | HR(95%CI) | P | HR(95%CI) | P |
| **Omega 3 Fatty Acids mmol/l** | 0.74(0.59,0.94) | 0.01 | 0.86(0.68,1.10) | 0.23 | 0.94(0.74,1.19) | 0.62 |
| **Omega 3 Fatty Acids Q** |  |  |  |  |  |  |
| Q1 | ref |  | ref |  | ref |  |
| Q2 | 0.91(0.79,1.05) | 0.19 | 0.96(0.84,1.10) | 0.57 | 0.99(0.86,1.14) | 0.87 |
| Q3 | 0.86(0.75,0.98) | 0.02 | 0.94(0.82,1.08) | 0.36 | 0.99(0.86,1.13) | 0.87 |
| **Omega 6 Fatty Acids mmol/l** |  |  |  |  |  |  |
| **Omega 6 Fatty Acids Q** | 0.81(0.75,0.88) | <0.0001 | 0.84(0.78,0.91) | <0.0001 | 0.88(0.81,0.95) | 0.001 |
| Q1 | ref |  | ref |  | ref |  |
| Q2 | 0.8(0.70,0.91) | <0.001 | 0.83(0.73,0.95) | 0.01 | 0.88(0.77,1.00) | 0.05 |
| Q3 | 0.74(0.65,0.84) | <0.0001 | 0.79(0.69,0.90) | <0.001 | 0.83(0.73,0.96) | 0.01 |
| **Omega 6/3 ratio** |  |  |  |  |  |  |
| **Omega 6/3 ratio Q** | 1.01(1.00,1.02) | 0.11 | 1(0.99,1.01) | 0.77 | 1(0.99,1.01) | 0.89 |
| Q1 | ref |  | ref |  | ref |  |
| Q2 | 1.02(0.90,1.16) | 0.75 | 0.99(0.87,1.12) | 0.88 | 0.98(0.86,1.11) | 0.71 |
| Q3 | 1.02(0.89,1.17) | 0.79 | 0.95(0.82,1.09) | 0.43 | 0.92(0.80,1.06) | 0.23 |

Abbreviations: BMI, body mass index; TDI, Townsend deprivation index; PRS, polygenic risk score; AD, Alzheimer's disease; HR, hazard ratios; CI, confidence intervals.

Crude model was adjusted for no covariates. Model 1 was adjusted for gender, ethnicity, education, TDI; Model 2 was additionally adjusted for household income, BMI, alcohol drinking status, smoking status, physical activity level, hypertension disease, DM, stroke.

Supplementary Table S12. Sensitive analysis 5:the association between omega-3 and omega-6 fatty acids and dementia risk with mutual adjustment of omega-3, omega-6, and AD PRS in cox regression analysis(N=81827)

| **Character** | **Crude model** | | **Model 1** | | **Model 2** | |
| --- | --- | --- | --- | --- | --- | --- |
|  | HR(95%CI) | P | HR(95%CI) | P | HR(95%CI) | P |
| **Omega 3 Fatty Acids mmol/l** | 1.68(1.36,2.09) | <0.0001 | 0.89(0.70,1.13) | 0.33 | 1.13(0.88,1.44) | 0.35 |
| **Omega 3 Fatty Acids Q** |  |  |  |  |  |  |
| Q1 | ref |  | ref |  | ref |  |
| Q2 | 1.14(1.00,1.31) | 0.06 | 0.96(0.83,1.10) | 0.55 | 1.02(0.89,1.18) | 0.76 |
| Q3 | 1.38(1.21,1.57) | <0.0001 | 0.95(0.83,1.09) | 0.44 | 1.08(0.94,1.25) | 0.29 |
| p for trend |  | <0.0001 |  | 0.45 |  | 0.98 |
| **Omega 6 Fatty Acids mmol/l** | 0.78(0.72,0.84) | <0.0001 | 0.82(0.76,0.89) | <0.0001 | 0.82(0.75,0.89) | <0.0001 |
| **Omega 6 Fatty Acids Q** |  |  |  |  |  |  |
| Q1 | ref |  | ref |  | ref |  |
| Q2 | 0.73(0.64,0.83) | <0.0001 | 0.8(0.70,0.91) | <0.001 | 0.83(0.73,0.95) | 0.01 |
| Q3 | 0.72(0.63,0.82) | <0.0001 | 0.76(0.66,0.87) | <0.0001 | 0.76(0.66,0.88) | <0.001 |
| p for trend |  | <0.0001 |  | <0.0001 |  | 0.002 |

Abbreviations: BMI, body mass index; TDI, Townsend deprivation index; PRS, polygenic risk score; AD, Alzheimer's disease; HR, hazard ratios; CI, confidence intervals.

Crude model was adjusted for no covariates. Model 1 was adjusted for gender, ethnicity, education, TDI; Model 2 was additionally adjusted for household income, BMI, alcohol drinking status, smoking status, physical activity level, hypertension disease, DM, stroke, AD PRS and Omega 3 Fatty Acids mmol/l (or Omega 6 Fatty Acids mmol/l ) differ for the exposure.

Supplementary Table S13. Sensitive analysis 6:the association between omega-3 and omega-6 fatty acids and dementia risk with aged more than 60 years old(Event=1130,N=28691)

| **Character** | **Crude model** | | **Model 1** | | **Model 2** | |
| --- | --- | --- | --- | --- | --- | --- |
|  | HR(95%CI) | P | HR(95%CI) | P | HR(95%CI) | P |
| **Omega 3 Fatty Acids mmol/l** | 0.74(0.57,0.95) | 0.02 | 0.85(0.66,1.10) | 0.22 | 0.91(0.70,1.17) | 0.45 |
| **Omega 3 Fatty Acids Q** |  |  |  |  |  |  |
| Q1 | ref |  | ref |  | ref |  |
| Q2 | 0.91(0.78,1.05) | 0.20 | 0.97(0.83,1.13) | 0.69 | 0.99(0.85,1.15) | 0.90 |
| Q3 | 0.83(0.72,0.96) | 0.01 | 0.92(0.79,1.07) | 0.26 | 0.96(0.82,1.11) | 0.56 |
| p for trend |  | 0.01 |  | 0.25 |  | 0.55 |
| **Omega 6 Fatty Acids mmol/l** | 0.75(0.69,0.82) | <0.0001 | 0.82(0.75,0.90) | <0.0001 | 0.84(0.77,0.92) | <0.001 |
| **Omega 6 Fatty Acids Q** |  |  |  |  |  |  |
| Q1 | ref |  | ref |  | ref |  |
| Q2 | 0.71(0.62,0.82) | <0.0001 | 0.79(0.69,0.91) | 0.001 | 0.82(0.71,0.95) | 0.01 |
| Q3 | 0.65(0.56,0.74) | <0.0001 | 0.74(0.63,0.85) | <0.0001 | 0.77(0.66,0.89) | <0.001 |
| p for trend |  | <0.0001 |  | <0.0001 |  | <0.001 |
| **Omega 6/3 ratio** | 1.01(0.99,1.02) | 0.42 | 1(0.99,1.02) | 0.73 | 1(0.99,1.01) | 0.97 |
| **Omega 6/3 ratio Q** |  |  |  |  |  |  |
| Q1 | ref |  | ref |  | ref |  |
| Q2 | 0.97(0.85,1.11) | 0.65 | 0.96(0.84,1.10) | 0.53 | 0.95(0.83,1.08) | 0.42 |
| Q3 | 1(0.86,1.16) | 1.00 | 0.96(0.83,1.12) | 0.62 | 0.94(0.81,1.09) | 0.43 |
| p for trend |  | 0.93 |  | 0.57 |  | 0.39 |

Abbreviations: BMI, body mass index; TDI, Townsend deprivation index; PRS, polygenic risk score; AD, Alzheimer's disease; HR, hazard ratios; CI, confidence intervals.

Crude model was adjusted for no covariates. Model 1 was adjusted for age, gender, ethnicity, education, TDI; Model 2 was additionally adjusted for household income, BMI, alcohol drinking status, smoking status, physical activity level, hypertension disease, DM, stroke.

Supplementary Table S14. Sensitive analysis 7:the association between omega-3 and omega-6 fatty acids and dementia risk with excluding all missing data(Event=500,N=46723)

| **Character** | **Crude model** | | **Model 1** | | **Model 2** | |
| --- | --- | --- | --- | --- | --- | --- |
|  | HR(95%CI) | P | HR(95%CI) | P | HR(95%CI) | P |
| **Omega 3 Fatty Acids mmol/l** | 2.12(1.50,2.98) | <0.0001 | 0.92(0.63,1.35) | 0.67 | 1.05(0.72,1.53) | 0.81 |
| **Omega 3 Fatty Acids Q** |  |  |  |  |  |  |
| Q1 | ref |  | ref |  | ref |  |
| Q2 | 1.3(1.03,1.64) | 0.02 | 1.02(0.81,1.28) | 0.87 | 1.07(0.85,1.36) | 0.54 |
| Q3 | 1.61(1.29,2.00) | <0.0001 | 0.99(0.79,1.25) | 0.95 | 1.08(0.86,1.36) | 0.53 |
| p for trend |  | <0.0001 |  | 0.92 |  | 0.55 |
| **Omega 6 Fatty Acids mmol/l** | 0.77(0.67,0.87) | <0.0001 | 0.78(0.68,0.90) | <0.001 | 0.83(0.73,0.95) | 0.01 |
| **Omega 6 Fatty Acids Q** |  |  |  |  |  |  |
| Q1 | ref |  | ref |  | ref |  |
| Q2 | 0.74(0.60,0.92) | 0.01 | 0.78(0.63,0.97) | 0.02 | 0.85(0.68,1.05) | 0.13 |
| Q3 | 0.7(0.57,0.87) | 0.001 | 0.7(0.56,0.87) | 0.001 | 0.76(0.60,0.95) | 0.02 |
| p for trend |  | <0.001 |  | 0.001 |  | 0.02 |
| **Omega 6/3 ratio** | 0.93(0.91,0.96) | <0.0001 | 0.99(0.97,1.02) | 0.63 | 0.99(0.97,1.01) | 0.38 |
| **Omega 6/3 ratio Q** |  |  |  |  |  |  |
| Q1 | ref |  | ref |  | ref |  |
| Q2 | 0.73(0.60,0.89) | 0.002 | 0.98(0.80,1.20) | 0.84 | 0.97(0.79,1.19) | 0.77 |
| Q3 | 0.49(0.39,0.61) | <0.0001 | 0.89(0.71,1.12) | 0.33 | 0.86(0.68,1.08) | 0.19 |
| p for trend(character2integer) |  | <0.0001 |  | 0.35 |  | 0.21 |

Abbreviations: BMI, body mass index; TDI, Townsend deprivation index; PRS, polygenic risk score; AD, Alzheimer's disease; HR, hazard ratios; CI, confidence intervals.

Crude model was adjusted for no covariates. Model 1 was adjusted for age, gender, ethnicity, education, TDI; Model 2 was additionally adjusted for household income, BMI, alcohol drinking status, smoking status, physical activity level, hypertension disease, DM, stroke.

Supplementary Table S15. Sensitive analysis 8: the association between omega-3 and omega-6 fatty acids and Alzheimer’s Disease risk (Event=611,N=81851)

| **Character** | **Crude model** | | **Model 1** | | **Model 2** | |
| --- | --- | --- | --- | --- | --- | --- |
|  | HR(95%CI) | P | HR(95%CI) | P | HR(95%CI) | P |
| **Omega 3 Fatty Acids mmol/l** | 2.1(1.55,2.85) | <0.0001 | 1.04(0.74,1.46) | 0.84 | 1.07(0.76,1.51) | 0.69 |
| **Omega 3 Fatty Acids Q** |  |  |  |  |  |  |
| Q1 | ref |  | ref |  | ref |  |
| Q2 | 1.23(1.00,1.52) | 0.05 | 1(0.81,1.23) | 0.99 | 1.02(0.83,1.26) | 0.85 |
| Q3 | 1.65(1.35,2.01) | <0.0001 | 1.07(0.87,1.31) | 0.54 | 1.09(0.89,1.34) | 0.39 |
| p for trend |  | <0.0001 |  | 0.51 |  | 0.37 |
| **Omega 6 Fatty Acids mmol/l** | 0.9(0.80,1.01) | 0.07 | 0.92(0.82,1.03) | 0.15 | 0.91(0.81,1.03) | 0.14 |
| **Omega 6 Fatty Acids Q** |  |  |  |  |  |  |
| Q1 | ref |  | ref |  | ref |  |
| Q2 | 0.83(0.68,1.00) | 0.05 | 0.89(0.73,1.08) | 0.24 | 0.89(0.73,1.09) | 0.27 |
| Q3 | 0.87(0.72,1.05) | 0.15 | 0.87(0.72,1.07) | 0.18 | 0.87(0.71,1.07) | 0.19 |
| p for trend |  | 0.14 |  | 0.18 |  | 0.19 |
| **Omega 6/3 ratio** | 0.94(0.92,0.97) | <0.0001 | 1(0.98,1.02) | 0.87 | 1(0.97,1.02) | 0.63 |
| **Omega 6/3 ratio Q** |  |  |  |  |  |  |
| Q1 | ref |  | ref |  | ref |  |
| Q2 | 0.68(0.57,0.82) | <0.0001 | 0.89(0.73,1.07) | 0.20 | 0.88(0.73,1.06) | 0.17 |
| Q3 | 0.52(0.43,0.64) | <0.0001 | 0.89(0.73,1.09) | 0.28 | 0.87(0.71,1.06) | 0.17 |
| p for trend |  | <0.0001 |  | 0.22 |  | 0.13 |
| **AD PRS** | 1.9(1.78,2.03) | <0.0001 | 1.94(1.82,2.08) | <0.0001 | 1.95(1.82,2.08) | <0.0001 |
| **AD PRS Q** |  |  |  |  |  |  |
| Low | ref |  | ref |  | ref |  |
| Intermediate | 1.52(1.14,2.02) | 0.005 | 1.54(1.16,2.06) | 0.003 | 1.55(1.16,2.07) | 0.003 |
| High | 5.31(3.99,7.07) | <0.0001 | 5.54(4.16,7.37) | <0.0001 | 5.57(4.18,7.41) | <0.0001 |
| p for trend |  | <0.0001 |  | <0.0001 |  | <0.0001 |

Abbreviations: BMI, body mass index; TDI, Townsend deprivation index; PRS, polygenic risk score; AD, Alzheimer's disease; HR, hazard ratios; CI, confidence intervals.

Crude model was adjusted for no covariates. Model 1 was adjusted for age, gender, ethnicity, education, TDI; Model 2 was additionally adjusted for household income, BMI, alcohol drinking status, smoking status, physical activity level, hypertension disease, DM, stroke.
